# Analysis of the platypus genome suggests a transposon origin for mammalian imprinting

**DOI:** 10.1186/gb-2009-10-1-r1

**Published:** 2009-01-02

**Authors:** Andrew J Pask, Anthony T Papenfuss, Eleanor I Ager, Kaighin A McColl, Terence P Speed, Marilyn B Renfree

**Affiliations:** 1 Department of Zoology, The University of Melbourne, Melbourne, Victoria 3010, Australia; 2 Department of Molecular and Cellular Biology, The University of Connecticut, Storrs, CT 06269, USA; 3 Bioinformatics Division, The Walter and Eliza Hall Institute, 1G Royal Parade, Parkville, Victoria 3050, Australia

## Abstract

**Background:**

Genomic imprinting is an epigenetic phenomenon that results in monoallelic gene expression. Many hypotheses have been advanced to explain why genomic imprinting evolved in mammals, but few have examined how it arose. The host defence hypothesis suggests that imprinting evolved from existing mechanisms within the cell that act to silence foreign DNA elements that insert into the genome. However, the changes to the mammalian genome that accompanied the evolution of imprinting have been hard to define due to the absence of large scale genomic resources between all extant classes. The recent release of the platypus genome has provided the first opportunity to perform comparisons between prototherian (monotreme; which appear to lack imprinting) and therian (marsupial and eutherian; which have imprinting) mammals.

**Results:**

We compared the distribution of repeat elements known to attract epigenetic silencing across the entire genome from monotremes and therian mammals, particularly focusing on the orthologous imprinted regions. There is a significant accumulation of certain repeat elements within imprinted regions of therian mammals compared to the platypus.

**Conclusions:**

Our analyses show that the platypus has significantly fewer repeats of certain classes in the regions of the genome that have become imprinted in therian mammals. The accumulation of repeats, especially long terminal repeats and DNA elements, in therian imprinted genes and gene clusters is coincident with, and may have been a potential driving force in, the development of mammalian genomic imprinting. These data provide strong support for the host defence hypothesis.

## Abstract 2

Comparisons between the platypus and eutherian mammalian genomes provides new insights into how epigenetic imprinting may have evolved in mammalian genomes.

## Background

Genomic imprinting is an epigenetic phenomenon that results in monoallelic gene expression. Amongst mammals, it has only been identified in the therians (marsupials and eutherians). Many hypotheses have been advanced to explain why genomic imprinting evolved in mammals, but few have examined how it arose [1]. The retention of genomic imprinting must confer an evolutionary advantage since the resulting haploinsufficiency is frequently associated with increased susceptibility to disease [2]. The most widely accepted hypothesis to explain why mammalian imprinting may have been retained is the 'kinship hypothesis' [3,4]. This suggests that imprinting evolved to regulate nutrient exchange between the mother and the developing fetus [4]. Indeed, almost all the imprinted genes identified thus far are widely expressed in the eutherian placenta [5], a primary site of nutrient exchange. Genomic imprinting is, therefore, thought to be absent in the egg-laying monotremes, as it is in other egg laying, non-mammalian amniotes [6], where maternal-fetal nutrient exchange is minimal. Furthermore, investigations of four imprinted therian genes have failed to detect any evidence for genomic imprinting in the monotremes: *IGF2 *[6], *IGF2R *[7] and *UBE3A *[8] are biallelically expressed in the platypus while *PEG10 *[9] is absent.

Until now, no one has been able to examine at the genome level how imprinting may have evolved due to the absence of large scale genomic resources available for all classes of mammals. Genomic imprinting may have evolved from the same mechanisms that silence transposable elements and invading foreign DNA within the genome. This is referred to as the host defence hypothesis [10] and is supported by the observation that most imprinted genes in eutherians are associated with repeat sequences and endogenous retroviruses [11,12]. The recently sequenced platypus genome [13] provides the key resource to examine how imprinting evolved, since it is thought to have arisen after the divergence of this group from the therian mammals. While a number of imprinted gene orthologues have now been mapped in the platypus [14], with the exception of the *DLK1 *locus [15], there has been limited detailed analyses of their surrounding genomic context.

Comparative analyses of the *PEG10 *locus between therian mammals, the platypus and chicken provided the first evidence that retrotransposition is directly involved in the acquisition of genomic imprinting [9]. Insertion of *PEG10 *in the therian genome was coincident with its differential methylation, established by host defence mechanisms. This was then selected for and maintained in the therian genome [9]. The host defence hypothesis predicts that an accumulation of foreign DNA elements would have occurred in all imprinted regions in therians. To gain a greater understanding of how imprinted regions have evolved and to comprehensively test the host defence hypothesis, we have examined, on a genome scale, the conservation of synteny and accumulation of repeats and retrotransposed elements within therian-imprinted regions by comparison with the entire platypus genome.

## Results

### Imprinted region conservation

**Figure 1:**
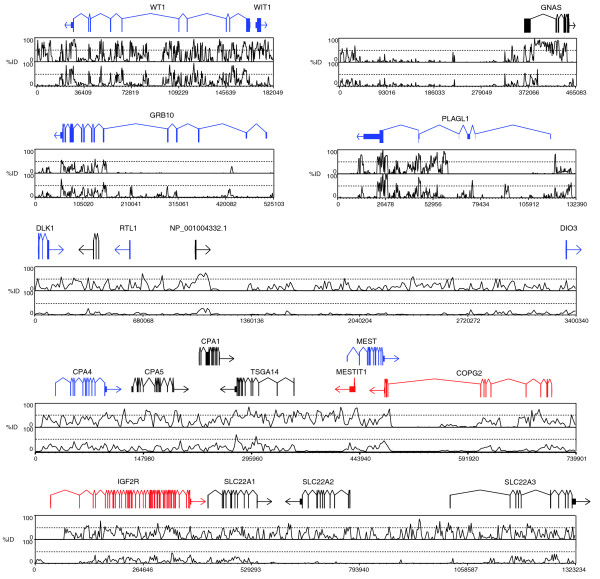
**Sequence conservation for seven of the investigated platypus regions orthologous to imprinted regions in human**. Each region shown is syntenic in the platypus. The top line shows the gene structure of the region (exons represented by boxes, introns by connecting lines). Human imprint status for the genes is indicated, with paternally imprinted genes shown in blue, maternally imprinted genes shown in red and non-imprinted genes or unknown status shown in black. The percentage identity (%ID) plot over the region for human compared to dog (top panel) and platypus (bottom panel) is shown beneath the gene structure for each region. As expected, conservation is higher between human and dog than human and platypus, reflecting their divergence times. The gene coding sequences are the most highly conserved, with little or no conservation found within intronic sequences. While most regions show a high degree of conservation, there is little seen throughout the *DIO3*/*DLK *cluster.

To determine imprinted gene conservation, we identified orthologous regions for all known eutherian imprinted genes across several mammalian species (n = 19 regions, encompassing 131 genes; Additional data file 4). We then examined orthologous sequences for all therian imprinted genes or regions that could be identified in the platypus genome (a subset is graphically represented in Figure 1, representing eutherian imprinted genes that are isolated (a single imprinted gene within a non-imprinted region) or in small or large imprinted clusters (two or more imprinted genes in close association)). We then determined the gene arrangement and sequence conservation of each orthologous region (Figure 1). In cases where the platypus was uninformative, due to incomplete assembly, the ancestral gene arrangement was confirmed by comparisons to the chicken genome. Orthologous sequences of the regions examined from human (NCBI 36), mouse (NCBI m36), dog (CanFam 2.0), opossum (MonDom5), platypus (OrnAna 5.0.1) and chicken (WASHUC2) were identified using gene orthology relationships from Ensembl (Release 44) [16]. Multiple alignments of each region were constructed using MLAGAN with translated anchoring [17]. Where syntenic regions in opossum or platypus were not contiguous or not assembled into a single sequence, the fragments were concatenated (with 60 'N's inserted between regions) for the purpose of alignment. This analysis confirmed that eutherian imprinted clusters are not recent assemblages, but instead reside in ancient syntenic mammalian groups. In some cases, these platypus regions lacked genes that have arisen specifically in the therians by mechanisms such as gene duplication or retrotransposition. Across all regions and species examined, sequence conservation was highest within the protein coding portions. The majority of intronic sequences showed little to no conservation across all species. However, there were some intronic regions that had high levels of sequence conservation, which may reflect non-coding RNAs, unannotated coding regions, or gene regulatory or enhancer elements (Figure 1).

### Repeat distribution across the entire genome and in orthologous imprinted regions

**Figure 2:**
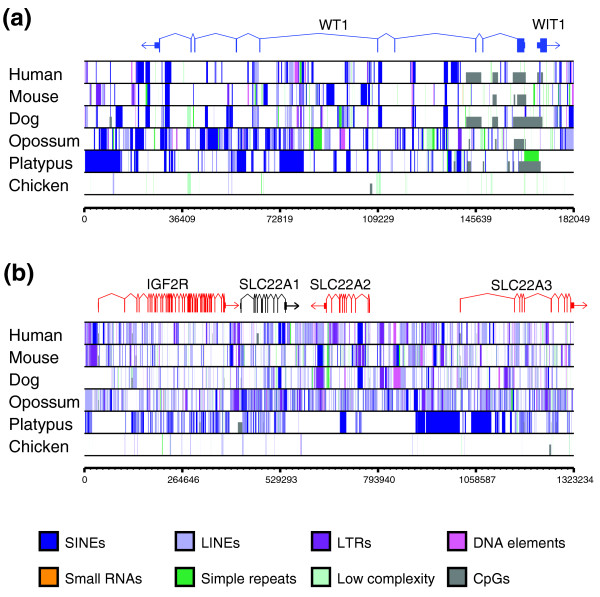
**Comparison of the spatial distribution of repeats for (a) an imprinted gene that is not in a cluster (*WT1*) and (b) an imprinted cluster (*IGF2R*) across the species examined in our analysis**. The repeat element distribution is shown as color-coded vertical lines in tracks for each species. There is a dramatic change in the number and spatial distribution of repeats between eutherians and platypus. There is also an increase in the number and size of CpG islands throughout the regions examined. Large-scale accumulation of repeats appears to have occurred after the bird-mammal divergence, with significantly fewer repeats of all classes seen in the chicken. The repeat distribution across an additional five regions of various sizes can be viewed in Additional data file 1. LINE, long interspersed nuclear element.

**Figure 3:**
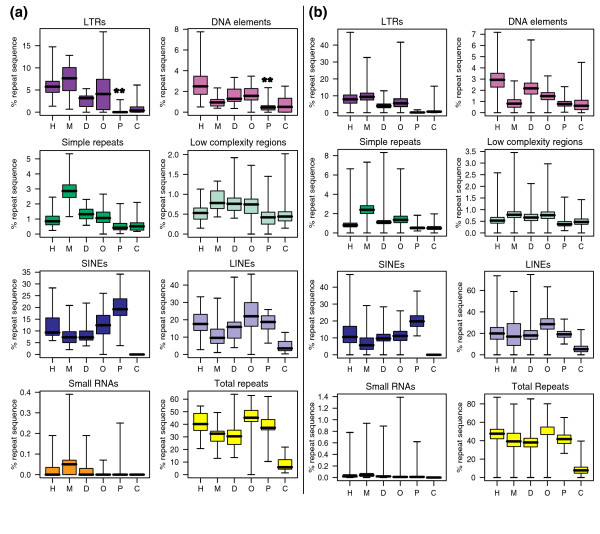
**Box-and-whiskers plot of the percent of sequence in each class of repeat element in (a) imprinted clusters (n = 19 covering 131 genes) and (b) the entire genome of each species**. Repeat sequences that had significantly different proportions in the platypus from all therian genomes are marked by double asterisks. Boxes indicate the interquartile range with whiskers showing the full range for each data set. Black lines within boxes indicate the median value. There are significantly fewer LTR and DNA elements across the platypus orthologous imprinted regions compared to all other mammalian species. Chicken has noticeably fewer total repeats and no SINEs or small RNAs. C, chicken; D, dog; H, human; M, mouse; O, opossum; P, platypus. LINE, long interspersed nuclear element.

We then examined the distribution of repeat elements known to attract silencing by host defence mechanisms (long interspersed nuclear element, short interspersed nuclear elements (SINEs), long terminal repeats (LTRs), low complexity and simple repeats and small non-coding RNAs) across the entire genome and within regions that are orthologous to eutherian imprinted regions (n = 19 regions, encompassing 131 genes; Figure 2; Additional data file 4) [13]. A summary of the repeat analysis across the orthologous gene clusters is presented in Figure 3a, and across the entire genome in Figure 3b (the proportion of repeats for each individual gene cluster is shown in detail in Additional data file 2a, b; the statistical analysis of these data is shown in Additional data file 5a, b). In the orthologous imprinted regions examined, the total proportion of sequence located in repeats of all types was not significantly different between platypus and other species (Figure 3a). However, the proportion of some specific repeat elements differed significantly between the monotremes and therian mammals (Additional data file 5). There were significantly fewer LTR elements (*p *≤ 0.002) and DNA elements (*p *≤ 0.02) in the platypus compared to all therian species. Long interspersed nuclear elements (*p *≤ 1), small RNAs (*p *≤ 1) and low complexity repeats (*p *≤ 1) were not significantly different across all regions in the platypus compared to other species. The proportion of SINEs in the platypus was significantly higher when compared to orthologous regions in eutherians (*p *≤ 0.02), but not with opossum (*p *= 0.06). However, this SINE increase is not unique to imprinted regions, but the result of the higher average SINE content of the platypus genome (20%) compared to eutherian mammals (8-13%) [13,18]. In contrast, the chicken had noticeably fewer total repeats and no SINEs or small RNAs, suggesting that the accumulation of these elements is a feature of the mammalian genome (Figure 3a). Repeat distribution analyses throughout the entire genomes of the species examined (Figure 3b; Additional data file 5a, b) demonstrate that the repeat accumulation is not restricted to the orthologous regions examined, but is a feature of the genomes as a whole. However, this analysis is at too coarse a level to identify specific and possibly small changes that can result in the acquisition of imprinting, such as the insertion of a single retrotransposon at the *PEG10 *locus [9].

### CpG island distribution

In the eutherians, the predominant mechanism of gene silencing is due to differential methylation of CpG islands, located in or near imprinted genes [19-21]. Therefore, we also examined the distribution of CpG islands within the orthologous imprinted clusters from all species (Figure 2; Additional data file 1). Given the overall high G-C content of the platypus genome compared to that of other mammals (45.5% in platypus versus 40% in eutherians [13]), it is surprising that the platypus gene clusters have relatively few CpG islands compared to all other mammalian species. This suggests that, in addition to an increase in repeat elements, the accumulation of CpG islands was also coincident with the acquisition of imprinting in the therian mammals and may have evolved as a secondary mechanism to stabilize the silencing mechanism.

## Discussion

Our platypus genome analyses have confirmed that eutherian imprinted clusters are not recent assemblages, but instead reside in ancient syntenic mammalian groups, as previously suggested (based on analysis of orthologues of eight imprinted genes in the platypus) [14]. In fact, the arrangement of most clusters appears to predate the divergence of birds and mammals as shown by the analysis of 61 genes over 12 clusters in non-mammalian vertebrate genomes [22]. Despite the conservation of gene arrangement within most orthologous imprinted gene clusters between all species examined, the regions have expanded greatly in the therian mammals compared to the platypus and chicken (Additional data file 3). This is particularly noticeable in the *IGF2 *and *SDHD *imprinted regions (Additional data file 2a, b), which show a rapid expansion in the therian mammals after divergence from the monotremes. The *IGF2 *region is the best-characterised imprinted domain among the mammals and is imprinted in both marsupials and eutherian mammals [1], but not in the monotremes [6]. The expansion of repeat classes within this cluster unequivocally coincides with the acquisition of imprinting to this region.

Analysis of the change in copy number of specific repeat classes showed that the platypus genome has significantly fewer LTRs and DNA elements within the gene clusters that became imprinted in the therian mammals. We suggest that the accumulation of LTRs and DNA elements in the therian genome is coincident with, and may have been the driving force in, the development of mammalian genomic imprinting.

LTRs comprise a particularly interesting class of repeat, as they are almost entirely absent from the platypus genome. Likewise, DNA elements are substantially lower in most, but not all, orthologous imprinted regions in the platypus and throughout the entire genome.

While the change in the incidence of repeats between platypus and therian mammals is only significant for LTRs and DNA elements across all regions combined, examination of each region individually indicates significant changes in other repeat classes within several specific regions. For example, the *GNAS *locus in eutherians has levels of DNA elements that are well below those in the platypus. However, the proportion of simple repeats for this region is dramatically higher in eutherians than in platypus. Similarly, low-complexity repeats are almost absent from the *RASGRF1 *locus in platypus, but increase rapidly in all other mammalian groups (Additional data file 2). This suggests that genomic imprinting may not be induced by a single class of repeat elements in all regions but rather an increase in any repeat type at a given locus.

Host defence mechanisms would also be attracted to repeats that move within the genome. However, our statistical analyses are limited to the detection of accumulation (insertion and expansion) of repeats but not their movement within clusters or the genome. The comparative spatial distribution of repeats (Additional data file 1) clearly demonstrates the different distribution of repeats within orthologous regions. Again, using the *GNAS *locus as an example, while the total percentage of SINEs is identical for both the platypus and human locus, in human the SINEs are distributed mainly within the *GNAS *gene, while in platypus they are found mainly within the 5' intergenic region. This could result from the movement of repeats or independent insertions in different lineages. Since our analyses are unable to discriminate between these events, our statistics are an underestimate of the changes occurring in the genome that may have attracted host defence silencing. A more detailed spatial examination of repeats could also help to explain the acquisition imprinting at some loci.

Whole genome repeat distribution analyses were also performed to determine if repeat expansion was a general feature of the mammalian genome or specific to just the orthologous imprinted regions. Our findings show that, as expected, repeat expansion is not restricted to certain regions, but a general feature of the mammalian genome. The random invasion and expansion of LTRs and DNA elements would have attracted host defence silencing mechanisms to many regions throughout the entire therian genome. This phenomenon would occasionally lead to the silencing of surrounding genes, resulting in a phenotypic effect. Only where this effect conferred an evolutionary advantage (in genes such as those that control fetal growth and maternal nutrient supply) would it have been selected for and maintained, creating an imprinted allele. This imprint can then spread to neighbouring genes, resulting in the characteristic clusters of silenced (or imprinted) genes in the genome. This suggestion is supported by the spread of imprinting observed in the *PEG10 *locus [9] and the rapid accumulation of repeat elements within the *IGF2 *imprinted gene cluster in therian mammals compared to the repeat deprived, non-imprinted orthologous domain in the platypus.

## Conclusion

This is the first complete analysis of the repeat distribution in the entire genome and imprinted clusters across all extant mammalian lineages. Since imprinting arose only in the viviparous (therian) mammals and is suggested to have occurred through the cooption of host defence mechanisms, comparisons of eutherian and marsupials with the newly available platypus genome provide the first opportunity for testing this hypothesis. Our findings provide strong, genome-wide support for the host defence hypothesis to explain the evolution of genomic imprinting in therian mammals. Our analyses show that the platypus has significantly fewer repeats of certain classes in the regions of the genome that have become imprinted in therian mammals. The accumulation of repeats, especially LTRs and DNA elements, is not specific to the orthologous imprinted regions but has occurred throughout the therian genome. Host defence mechanisms such as DNA methylation would have been attracted to silence newly inserted foreign elements. This occasionally led to the silencing (imprinting) of adjacent genes. This 'imprint' was selected for, and maintained where it conferred an evolutionary advantage - for example, in genes that had functions in fetal growth, placentation or nutrient exchange - leading to the evolution of mammalian genomic imprinting.

## Materials and methods

Repeat annotations were obtained for the human, mouse, dog, opossum, platypus and chicken genomes from the UCSC genome browser. The proportion of sequence in repetitive elements in imprinted gene clusters (including 20 kb flanking sequences) was then calculated. The proportion of sequence in repetitive elements was also calculated in 700 kb blocks across all genomes (this was the average size of imprinted clusters in human).

The repeats shown in Figure 2 were identified using RepeatMasker [23] for all species except platypus, where the whole-genome repeat analysis [13] was used. CpG islands (defined as more than 200 bp of continuous sequence with a C-G percentage greater than 60%) that attract methylation in imprinted regions in eutherian mammals were identified using a modified version of the CpGLH program by G Miklem and L Hillier [19].

Statistical analyses were performed using R [24]. For each repeat family, the proportion of sequence was transformed using:

$$p^{\prime }=\arcsin\left(\sqrt{p}\right)$$

To test for differences between the proportions of each repeat family in each species, all pairwise two-tailed *t*-tests were performed. The Holm method of correction for multiple testing was applied [25]. In all tests, n = 19 (gene clusters) and the significance level was α = 0.05. For comparisons between platypus and therians or eutherians, the *p*-values quoted in the text are the largest of the adjusted *p*-values for all tests between platypus and those species considered (therian or eutherian). Complete results are provided in Additional data file 5.

## Abbreviations

LTR: long terminal repeat; SINE: short interspersed nuclear element.

## Authors' contributions

AJP, ATP and MBR designed the study. ATP, KAM, TPS and EIA carried out the analyses, calculations and performed the statistical analyses. AJP, ATP and MBR prepared the manuscript. All authors read and approved the final manuscript.

## Additional data files

The following additional data are available with the online version of this paper. Additional data file 1 shows the comparison of the spatial distribution of repeats for seven of the regions examined in our analysis. Additional data file 2 shows the analysis of percent sequence comprised by each class of repeat element separated by each region. Additional data file 3 shows a comparative gene map of the *IGF2R *imprinted region. Additional data file 4 shows the conservation of imprinted gene orthologues and regions within the human, mouse, dog, opossum, platypus and chicken genomes. Additional data file 5 shows the adjusted *p*-values from all pairwise *t*-tests comparing the transformed proportion of sequence in each repeat class between each species for the 19 genes and regions shown in Additional data file 4 and throughout the entire genome.

## Supplementary Material

Additional data file 1 Comparison of the spatial distribution of repeats for seven of the regions examined in our analysis. Click here for file

Additional data file 2 Analysis of percent sequence comprised by each class of repeat element separated by each region. Click here for file

Additional data file 3 Comparative gene map of the *IGF2R *imprinted region. Click here for file

Additional data file 4 Conservation of imprinted gene orthologues and regions within the human, mouse, dog, opossum, platypus and chicken genomes. Click here for file

Additional data file 5 Adjusted *p*-values from all pairwise *t*-tests comparing the transformed proportion of sequence in each repeat class between each species for **(a) **the 19 genes and regions shown in Additional data file 4 and **(b) **throughout the entire genome. The Holm method of correction for multiple testing was used [25]. Significant results are underlined. Click here for file
