# Supplementary material for: Analysis of the platypus genome suggests a transposon origin for mammalian imprinting
Source: Genome Biol. 2009 Jan 2;10(1):R1. doi: 10.1186/gb-2009-10-1-r1 (PMC2687786; doi:10.1186/gb-2009-10-1-r1)
Supplement: Additional data file 1 — Comparison of the spatial distribution of repeats for seven of the regions examined in our analysis. [file gb-2009-10-1-r1-S1.pdf]

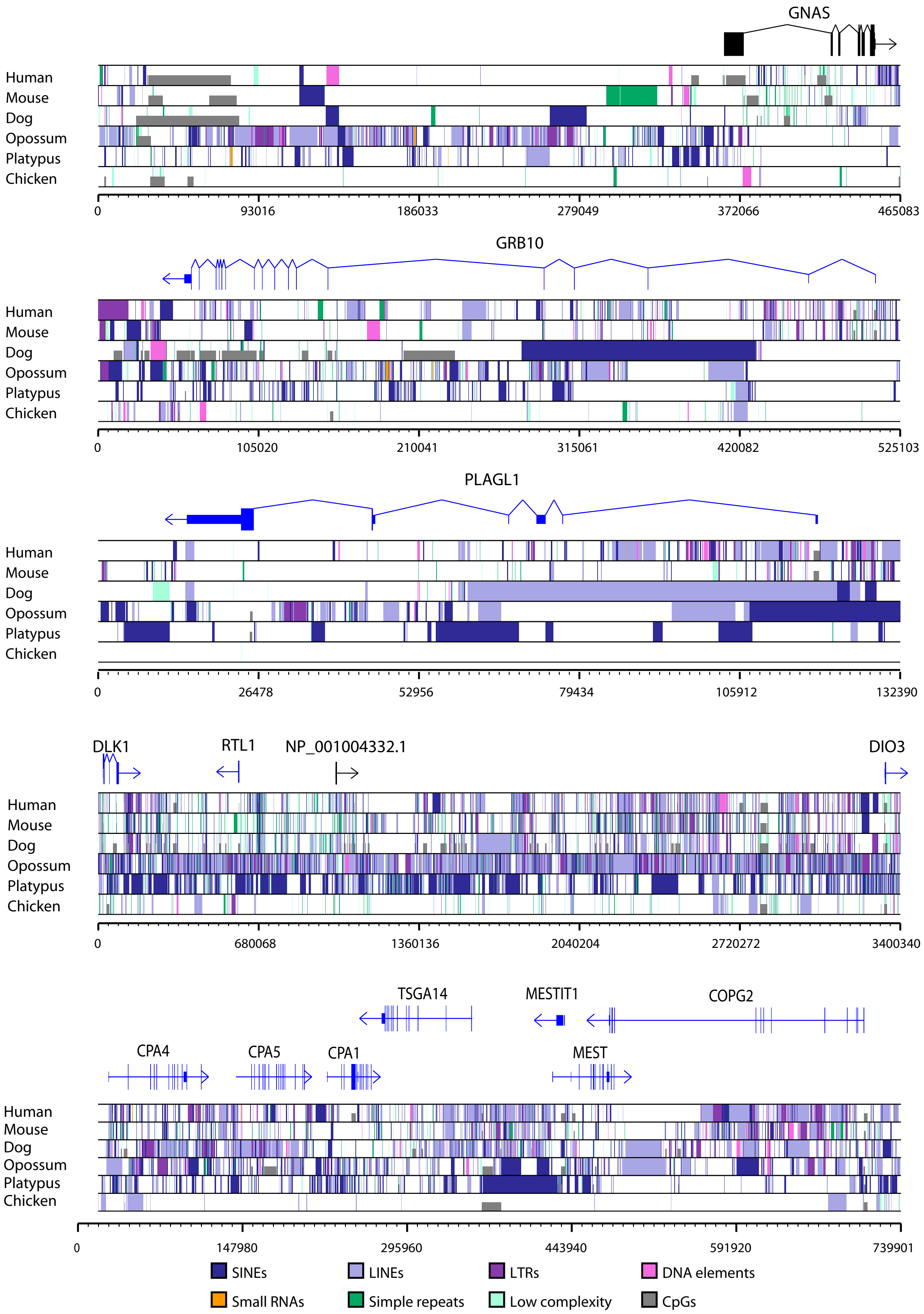


Additional Data File1: Comparison of the spatial distribution of repeats for seven of the regions examined in our analysis. The repeat element distribution is shown as colour coded vertical lines in tracks for each species. There was a dramatic change in the number and spatial distribution of repeats between therians and platypus. There was also an increase in the number and size of CpG islands throughout the regions examined. Large-scale accumulation of repeats appears to have occurred since the bird-mammal divergence with significantly fewer repeats of all classes seen in the chicken.
