# Supplementary material for: Analysis of the platypus genome suggests a transposon origin for mammalian imprinting
Source: Genome Biol. 2009 Jan 2;10(1):R1. doi: 10.1186/gb-2009-10-1-r1 (PMC2687786; doi:10.1186/gb-2009-10-1-r1)
Supplement: Additional data file 2 — Analysis of percent sequence comprised by each class of repeat element separated by each region. [file gb-2009-10-1-r1-S2.pdf]

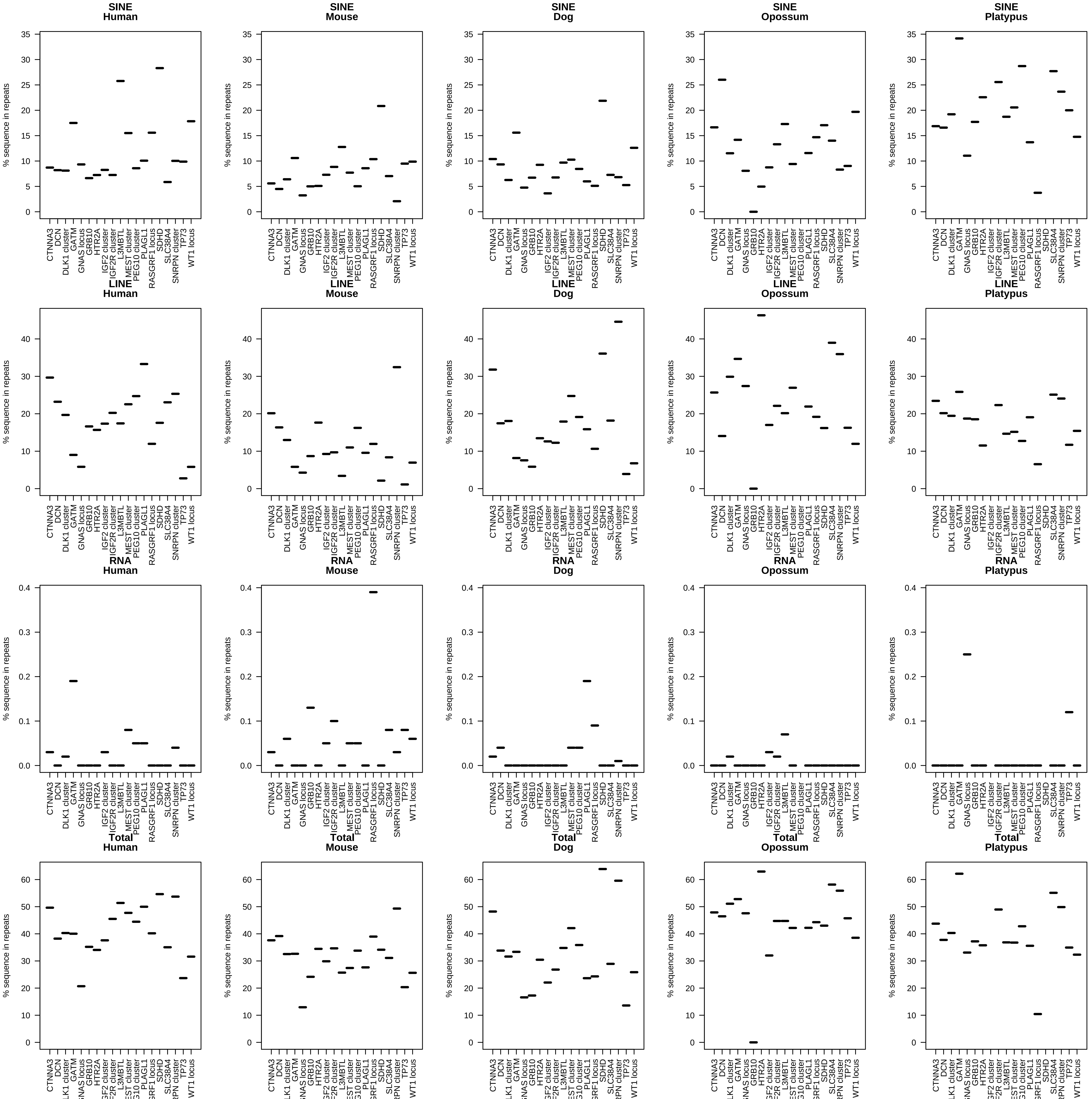

SINE Human
SINE Mouse
SINE Dog
SINE Opossum
SINE Platypus
LINE Human
LINE Mouse
LINE Dog
LINE Opossum
LINE Platypus
RNA Human
RNA Mouse
RNA Dog
RNA Opossum
RNA Platypus
Total Human
Total Mouse
Total Dog
Total Opossum
Total Platypus
% sequence in repeats
CTNNA3
DCN
DLK1 cluster
GATM
GNAS locus
GRB10
HTR2A
IGF2 cluster
IGF2R cluster
L3MBTL
MEST cluster
PEG10 cluster
PLAGL1
RASGRF1 locus
SDHD
SLC38A4
SNRPN cluster
TP73
WT1 locus

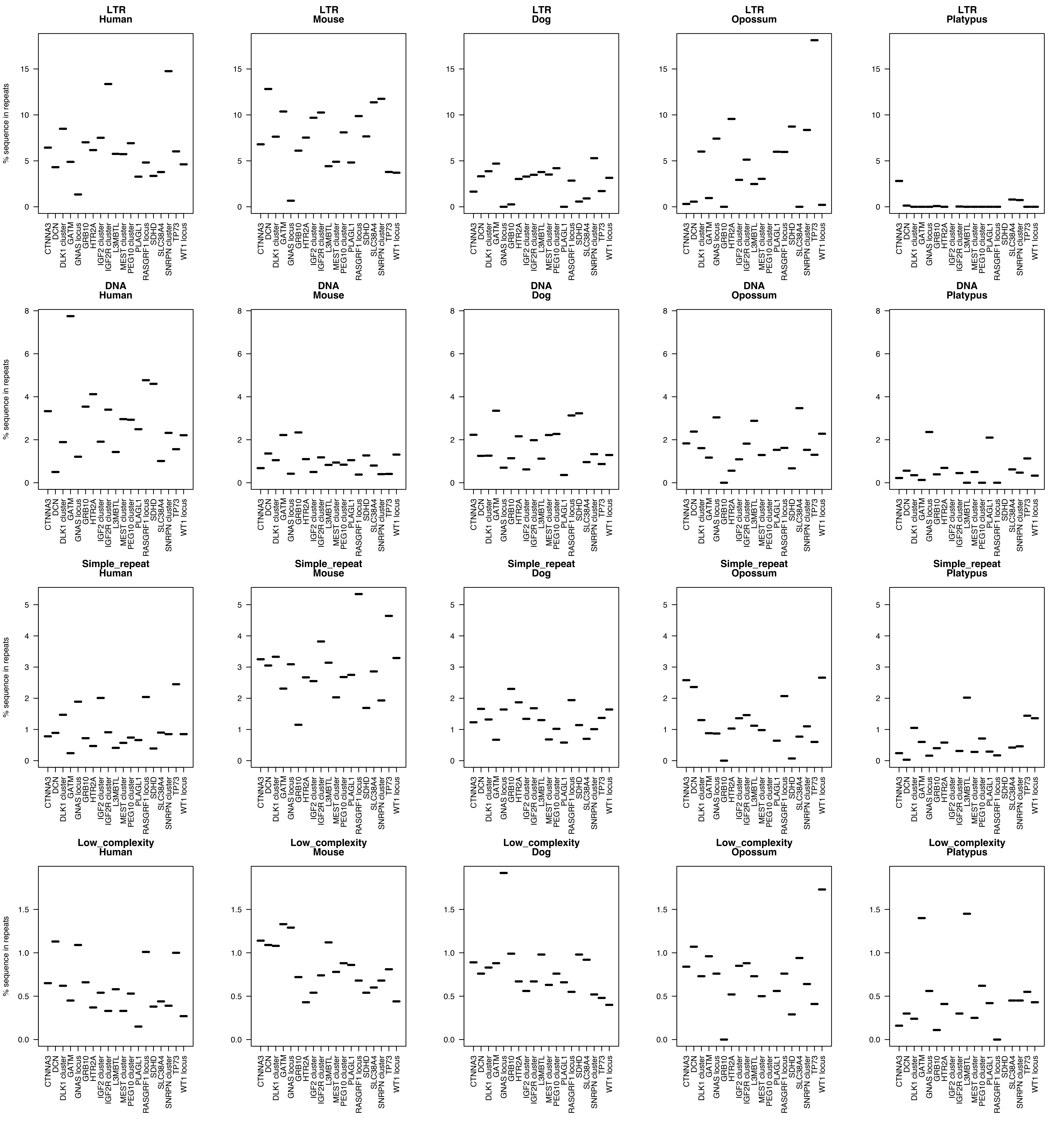

Additonal data file 2: Analysis of percent sequence comprised by each class of repeat element separated by each region (In alphabetical order, and as shown in yellow highlight, Additional Data file 4). The black lines indicate the percent sequence for each repeat class, within each cluster. A) When all regions were combined for analysis (Figure 3) only LTRs and DNA elements showed a significant change in percent sequence coverage between platypus and therian mammals. However, when each region was examined individually for repeats, there were clear differences between the non-significant repeat classes within specific regions. Simple repeats were noticeably lower in the GNAS and IGF2R regions of the platypus when compared with all therians. Similarly, low complexity repeats were noticeably lower in the GRB10 and DLK1 regions of the platypus compared to therians. B) Other repeat classes did not show any noticeable trends. This analysis also showed an unexpected increase in small RNAs that is unique to the platypus GNAS locus, suggesting this region may be predisposed to the accumulation of repeat elements.
