# Supplementary material for: Analysis of the platypus genome suggests a transposon origin for mammalian imprinting
Source: Genome Biol. 2009 Jan 2;10(1):R1. doi: 10.1186/gb-2009-10-1-r1 (PMC2687786; doi:10.1186/gb-2009-10-1-r1)
Supplement: Additional data file 3 — Comparative gene map of the IGF2R imprinted region. [file gb-2009-10-1-r1-S3.pdf]

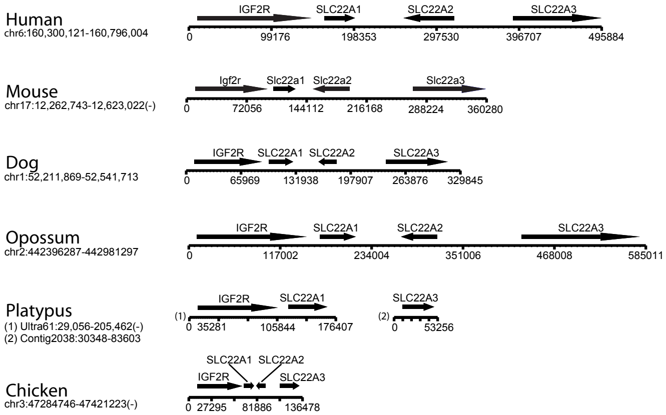

Additonal Data File 3: Comparative gene map of the IGF2R imprinted region. The arrows denote the location of genes within the region with the direction of transcription indicated by the arrowhead. Blue arrows indicate paternal imprinting, red indicates maternal imprinting and black indicated genes that are either not imprinted or the status is unknown. Numbers located beneath the line indicate base-pairs. While the gene order has remained highly conserved, the region has expanded in the eutherian mammals when compared to the platypus. This was also true for the MEST cluster (Warren et al. 2008). This is largely due to the accumulation of repetitive elements within the region that likely led to the acquisition of genomic imprinting. The orthologous platypus region resides on two contigs (labelled 1 and 2). However, since the chicken genome has the ancestral clustered organization of this region, it is presumed also to be syntenic in the platypus.
