# Supplementary material for: Analysis of the platypus genome suggests a transposon origin for mammalian imprinting
Source: Genome Biol. 2009 Jan 2;10(1):R1. doi: 10.1186/gb-2009-10-1-r1 (PMC2687786; doi:10.1186/gb-2009-10-1-r1)
Supplement: Additional data file 4 — Conservation of imprinted gene orthologues and regions within the human, mouse, dog, opossum, platypus and chicken genomes. [file gb-2009-10-1-r1-S4.pdf]

Map page	Chrom. Band (human)	Genes/features	Human	Mouse	Dog	Opossum	Platypus	Chicken
1	1p36.32	TP73	chr1:3,558,944-3,642,625 (I)	chr4:152,902,082-152,984,008 (I)	chr5:61,199,527-61,241,393	chr4:389,858,020-389,945,132	Ultra178:1,123,945-1,143,179	chr21:912,435-939,684
NA	1p31	ARHI/DIRAS3	chr1:68,283,023-68,290,278 (I)	NO	NO	NO	NO	NO
2	2p15	COMMD1<br>(U2af1-rs1)	chr2:61,986,292-62,216,698 (NI)<br>NO'	chr11:22,799,728-22,882,284 (I)<br>chr11:22,873,215-22,876,498 (I)	chr10:65,037,469-65,210,770<br>NO	chr1:630,290,345-630,540,770<br>NO	Ultra56:4,473,791-4,620,115<br>NO	chr3:8,903,451-8,969,942<br>NO
NA	4q13	*(Mkrn1-ps1)*	NO'	In the interval<br>chr5:89,857,838-90,00,9311 (I)	NA	NA	NA	NA
3	4q22	NAP1L5	chr4:89,837,380-89,837,928 (NR)	chr6:58,836,075-58,836,545 (I)	chr32:14,918,042-14,918,578	NO	NO	NO
4	6q24	PLAGL1<br>*HYMAI*	chr6:144,303,130-144,371,246 (I)<br>I	chr10:12,780,986-12,819,853 (I)	chr1:38,462,172-38,467,955	chr2:422,110,909-422,129,064	chr2:17,204,472-17,210,716	chr3:54,201,457-54,202,728
5	6q25	IGF2R cluster	chr6:160,310,121-160,796,004	chr17:12,263,330-12,613,022	chr1:52,221,869-52,539,630	chr2:442,406,287-442,973,871	Fragmented & most genes not in WGS assembly	chr3:47,288,282-47,411,223
		IGF2R	NI	I	O	O	Chr 2 - centric* Fragment on Ultra61	O
		(Air)	NO'	I	NA	NA	NA	NA
		SLC22A2	NR	I	O	O	Missing from WGS assembly	O
		SLC22A3	NR	I	O	O	Contig2038	O
6	7p12	GRB10	chr7:50,625,260-50,828,652 (I)	chr11:11,830,511-11,937,390 (I)	chr18:4,878,320-4,905,682	chr6:267,072,104-267,174,894	chr4:9,189,613-9,284,939 (chr 4p*)	chr2:83,096,709-83,234,014
7	7q21	PEG10 cluster	chr7:92,891,735-96,492,079	chr6:3,637,395-6,874,963	chr14:21,872,312-25,033,706	chrUn:10,910,032-84,197,983<br>Some are clustered to contigs.	The cluster is fragmented and most genes<br>are not present in the WGS assembly.	chr2:22,860,906-24,415,562
		CALCR	PD	I	O	NO	NO	NO
		SGCE	I	I	O	O	O (Contig1947)	O
		PEG10	I	I	NO	NO	NO	NO
		PPP1R9A	I	I	O	NO	NO	NO
		PON1	PD	NI	O	O	Not present	
		PON3	NR	PD	O	O	Not present	A single PON family member is present
		PON2	NR	PD	O	O	Not present	
		ASB4	NR	I	O	O	Not present	O
		DLX5	I	NI	O	O	Not present	O
8	7q32	MEST cluster	chr7:129,720,229-130,004,138	chr6:30,518,386-30,827,142	chr14:9,304,266-9,594,583	chr8:190,305,230-190,512,697	chr10:4,897,302-5,045,606	chr1:818,869-873,265
		CPA4	I	NR	O	O	O	P
		MEST	I	I	O	O	O	O
		MESTIT1	I	NO'	NA	NA	NA	NA
		COPG2IT1	I	I	NA	NA	NA	NA
		COPG2	CD	I	O	O	O	NO
NA	mouse 15qD3	*(Peg13)*	NO'	chr15:72,632,860-72,637,580 (I)	NA	NA	NA	NA
9	10q22	CTNNA3	chr10:67,349,725-69,125,933 (PD)	chr10:62,899,394-64,398,190 (NR)	chr4:20,577,292-20,755,158<br>chr4:22,057,498-22,195,110	chr1:29,149,665-29,267,868	chr3:54,029,573-54,043,119	chr6:7,775,606-8,203,040
NA	10q25	Ins1	NO'	chr19:52,317,765-52,318,343 (CD)	NO	NO	NO	NO
10	11p15	IGF2 cluster	chr11:1,972,984-3,143,116	chr7:142,244,367-143,587,755	chr18:49,099,550-50,068,310	The cluster is fragmented and several<br>genes are not present in the WGS.<br>TH is on chr8:82,441,041-82,502,283	None of the eutherian-imprinted genes<br>are present in the annotation.<br>Cluster is split across multiple contigs.<br>TH is on Ultra443.	chr5:14,090,506-14,883,396<br>Key orthologues are present & clustered.
		H19	I	I	NA	NA	NA	NA
		IGF2	I	I	O	NA	NA (Chr 2 centric*)	O
		IGF2AS	I	I	NA	NA	NA	NA
		INS	I	I	O	NA	NA	O
		ASCL2	CD	I	NO	NO	NO	NO
		PHEMX	NI	I	O	NO	*NO*	NO
		CD81	NI	I	O	NO	NO	NO
		TSSC4	NI	I	O	O (chrUn:76,673,683-76,674,915)	NO	O
		TRPM5	PD	NI	O	NO	NO	O
		KCNQ1	I	I	O	O (chrUn:76,673,683-76,674,915)	NO	O
		KCNQ1OT1	I	I	NA	NA	NA	NA
		KCNQ1DN	I	NO	NO	NO	NO	NO
		CDKN1C	I	I	NO	NO	NO	NO
		SLC22A1LS	PD	NO	NO	NO	NO	NO
		(Msuit1)	NO	I	NA	NA	NA	NA
		SLC22A18	I	I	O	NO	NO	O
		PHLDA2	I	I	NO	NO	O (Contig17670)	NO
		NAP1L4	NR	I	O	O (chr5:301647943-301923441)	NO	O

Map page	Chrom. Band (human)	Genes/features	Human	Mouse	Dog	Opossum	Platypus	Chicken
		(Tnfrsf23)	NO	I	NO	NO	NO	NO
		OSBPL5	I	I	O	O (chr5:301647943-301923441)	NO	O
		ZNF215	PD	NO	NO	NO	NO	NO
11	11p13	WT1 locus	chr11:32,365,901-32,413,663	chr2:104,927,368-104,974,451	chr18:38,119,539-38,167,266	chr5:268,104,216-268,165,744	chr3:18,288,268-18,331,210	chr5:5,499,297-5,531,098
		WT1	I	NR	O	O	O	O
		WT1AS	I	NR	NA	NA	NA	NA
12	11q23	SDHD	chr11:111,462,832-111,471,727 (CD)	chr9:50,348,576-50,356,047 (NR)	chr8:49,750,737-49,751,573	chr4:241,152,531-241,160,001	SDHS is missing from the annotation.<br>Syntenic region is fragmented on separate contigs.	chr24:6,297,764-6,300,662
13	12q13	SLC38A4	chr12:45,444,813-45,506,006 (NR)	chr15:96,822,857-96,883,990 (I)	chr27:10,739,997-10,766,280	chr8:122,487,624-122,506,648	chr2:53,474,030-53,504,705 (Chr 2q*)	chr1:32,895,397-32,911,123
14	12q21	DCN	chr12:90,063,167-90,100,937 (NR)	chr10:96,912,055-96,947,839 (I)	chr15:34,846,602-34,889,996	chr8:67,411,591-67,463,268	Ultra443:10,376,783-10,413,091	chr1:45,901,552-45,943,626.
15	13q14	HTR2A	chr13:46,305,514-46,368,179 (CD)	chr14:73,374,995-73,441,014 (I)	chr22:7,395,610-7,452,531	chr4:322,101,576-322,169,184	Ultra336:6,401,638-6,431,883	chr1:172,637,846-172,662,791
16	14q32	DLK1 cluster	chr14:100,262,917-101,099,540	chr12:109,901,030-110,727,953	chr8:72,005,936-72,740,082	chr1:31,586,115-317,465,410	Ultra378:6,295,837-6,330,250	chr5:51,436,508-51,458,293
		DLK1	I	I	O	O	O (Chr 1q*)	O
		DLK1 downstream transcripts	NR	I	NA	NA	NA	NA
		MEG3	I	I	NA	NA	NA	NA
		miR-337	NR	I	NA	NA	NA	NA
		RTL1	NR	I	O	NO	NO	NO
		Anti-PEG11	NR	I	NA	NA	NA	NA
		miR-127	NR	I	NA	NA	NA	NA
		miR-136	NR	I	NA	NA	NA	NA
		MEG8	NR	I	NA	NA	NA	NA
		miR-370	NR	I	NA	NA	NA	NA
		(MBII-78)	NO	I	NA	NA	NA	NA
		(MBII-19)	NO	I	NA	NA	NA	NA
		14q(0)	NR	I	NA	NA	NA	NA
		14q(I)/14q(II) region	NR	I	NA	NA	NA	NA
		(MBII-49)	NO	I	NA	NA	NA	NA
		(MBII-426)	NO	I	NA	NA	NA	NA
		(Mirg)	NR	I	NA	NA	NA	NA
		miR-411	NR	I	NA	NA	NA	NA
		miR-380	NR	I	NA	NA	NA	NA
		miR-376b	NR	I	NA	NA	NA	NA
		miR-376	NR	I	NA	NA	NA	NA
		miR-134	NR	I	NA	NA	NA	NA
		miR-154	NR	I	NA	NA	NA	NA
		miR-410	NR	I	NA	NA	NA	NA
		DIO3	NR	I	O	O	DIO3 is missing from the annotation. (Chr 1q*)	O
17	15q11	SNRPN cluster	chr15:21,161,547-25,651,622	chr7:56,592432-62,297,357	chr3:35,809,724-39,171,960	chr7:55,210,741-58,706,093	Cluster is split across ultracontigs in assembly.	chr1:58,845,921-136,000,661<br>Part of cluster only
		(Peg12)	NO	I	NO	NO	NO	NO
		MKRN3	I	I	O	O (chrUn:24,236,162-24,237,136)	NO	NO
		ZNF127AS	NR	I	NA	NA	NA	NA
		MAGEL2	I	I	NO	NO	NO	NO
		NDN	I	I	O	NO (2 paralogues)	NO	NO
		(Nccr)	NR	I	NO	NO	NO	NO
		SNURF	I	I	O	NO	NO	NO
		SNRPN	I	I	O	NO (SNRPB on chr1)	NO	NO (SNRPB on chr20)
		HBII-436	I	NR	NA	NA	NA	NA
		HBII-13	I	I	NA	NA	NA	NA
		HBII-437	I	NO	NA	NA	NA	NA
		HBII-438A	I	NO	NA	NA	NA	NA
		PWCR1	I	I	NA	NA	NA	NA
		HBII-52	I	I	NA	NA	NA	NA
		HBII-438B	I	NO	NA	NA	NA	NA
		UBE3A-AS	I	I	NA	NA	NA	NA
		UBE3A	I	I	O	O	O (Ultra222:9,229,418-9,254,470) (Chr 18p*)	O (chr1:58,845,921-136,000,661)
		ATP10A	I	CD	O	O	O (Ultra292:27,909-686,043)	O (chr1:58,845,921-136,000,661)
		GABRB3	CD	NR	O	O	O (Ultra292:27,909-686,043)	O (chr1:58,845,921-136,000,661)
		GABRA5	CD	NR	O	O	O (Ultra292:27,909-686,043)	O (chr1:58,845,921-136,000,661)
		GABRG3	CD	NR	O	O	O (Ultra292:27,909-686,043)	O (chr1:58,845,921-136,000,661)
18	15q21	GATM	chr15:43,440,616-43,457,943 (NR)	chr2:122,285,913-122,302,709 (I)	chr30:14,716,915-14,732,295	chr1:203,449,595-203,470,180	chr2:1,658,926-1,691,600	chr12:12,588,264-12,601,291
	15q24	RASGRF1 locus	chr15:77,041,541-77,170,161	chr9:89,624,837-89,824,819 (I)	chr3:60,858,345-60,950,268	chr1:120,874,179-121,090,120	Contig26251	chr10:22,105,786-22,124,409
19		*(4930524O08Rik/A19)*	NO	I	NA	NA	NA	NA

Map page	Chrom. Band (human)	Genes/features	Human	Mouse	Dog	Opossum	Platypus	Chicken
		RASGRF1	NR	I	O	O	O	O
20	18q11	IMPACT	chr18:20,260,680-20,287,490 (NI)	chr18:13,115,421-13,136,117 (I)	chr7:66,986,620-66,996,951	chr3:259,995,454-260,031,256	Contig20839	chr2:106,529,413-106,547,483
21	18q21	TCEB3C	chr18:42,808,571-42,810,211 (I)	NO	NO	NO	NO	NO
22	19q13		chr19:61,977,742-62,422,337	chr7:6281009-6600000	chr1:104,184,489-104,196,263	No conserved synteny	No conserved synteny	No conserved synteny
		PEG3/ZIM2	I	I	O	NO	NO	NO
		(Zim1)	NO	I	NO	NO	NO	NO
		ZIM2	I	I	NO	NO	NO	NO
		USP29	NR	I	NO	NO	NO	NO
		ZIM3	NR	I	NO	NO	NO	NO
		ZNF264	NR	I	NO	NO	NO	NO
23	20q11	NNAT	chr20:35,583,021-35,585,502 (I)	chr2:157,251,551-157,253,958 (I)	chr24:29,090,140-29,091,946	NO	NO	NO
24	20q13	L3MBTL	chr20:41,575,558-41,603,949 (I)	chr2:162,638,630-162,665,966 (NR)	chr24:34,211,452-34,235,635	chr1:385,894,559-385,957,809	Ultra337:4,152,550-4,172,414	chr20:29,872-47,955
25	20q13	GNAS locus	chr20:56,848,168-56,919,642	chr2:173,927,270-173,989,683	chr24:46,583,771-46,639,038	chr1:483,863,859-484,051,250	Ultra516:8,105,240-8,223,121	chr20:10,870,284-10,950,671
		GNAS	I	I	O	O	O (Chr 8p*)	O
		SANG (Nespas)	I	I	NA	NA	NA	NA
NA	Xq13	XIST locus	chrX:72,957,211-72,989,313	chrX:99,663,092-99,670,276				
		XIST	NI	I	NA	NA	NA	NA
		TSIX	NI	I	NA	NA	NA	NA

Additional Data file 4: Conservation of imprinted gene orthologues and regions within the human, mouse, dog, opossum, platypus and chicken genomes. The location of orthologous sequences is listed for each species. Localisations denoted with * are derived from reference Edwards et al. 2007. O indicates the presence of a gene orthologue, NO indicates no orthologue found, NO' indicates no orthologue found, based on previous analyses (Morison et al. 2005), NA indicates orthologues were not in the assembly/annotation, I indicates imprinted genes, NI indicated non-imprinted genes, NR indicated no reports of imprinting status, CD indicated conflicting data with respect to imprint status and PD indicated provisional data based on previous analyses (Morison et al. 2005). With the exception of the SNRPN locus, the genes that are conserved across all species, form ancient syntenic clusters. Some clusters have arisen recently and appear to be specific to the eutherians. Further detailed analyses of repeat distribution were performed on nineteen genes or regions shown from this analysis (highlighted in yellow) to be conserved across all genomes examined.
