# Supplementary material for: Analysis of the platypus genome suggests a transposon origin for mammalian imprinting
Source: Genome Biol. 2009 Jan 2;10(1):R1. doi: 10.1186/gb-2009-10-1-r1 (PMC2687786; doi:10.1186/gb-2009-10-1-r1)
Supplement: Additional data file 5 — Adjusted p-values from all pairwise t-tests comparing the transformed proportion of sequence in each repeat class between each species for (a) the 19 genes and regions shown in Additional data file 4 and (b) throughout the entire genome. The Holm method of correction for multiple testing was used [25]. Significant results are underlined. [file gb-2009-10-1-r1-S5.pdf]

p-values from pairwise t-tests comparing proportion of sequence in each repeat class between each species in orthologous imprinted regions

Additional data file 5

Repeat family		Human	Mouse	Dog	Opossum	Platypus
LTRs	Mouse	0.7	-	-	-	-
	Dog	0.01	0.001	-	-	-
	Opossum	0.4	0.2	0.4	-	-
	Platypus	6×10^{-9}	4×10^{-10}	0.002	6×10^{-6}	-
	Chicken	2×10^{-7}	2×10^{-8}	0.03	0.0002	0.7
DNA elements	Mouse	0.02	-	-	-	-
	Dog	0.08	0.97	-	-	-
	Opossum	0.2	0.97	1	-	-
	Platypus	7×10^{-5}	0.02	0.002	0.006	-
	Chicken	3×10^{-5}	0.006	0.001	0.002	1
Simple repeats	Mouse	7×10^{-7}	-	-	-	-
	Dog	0.4	2×10^{-5}	-	-	-
	Opossum	1	0.0004	1	-	-
	Platypus	0.4	1×10^{-7}	0.01	0.4	-
	Chicken	0.4	5×10^{-10}	0.0007	0.3	1
Low complexity	Mouse	0.08	-	-	-	-
	Dog	0.2	1	-	-	-
	Opossum	1	1	1	-	-
	Platypus	1	0.05	0.07	1	-
	Chicken	1	0.5	0.6	1	1
SINEs	Mouse	0.1	-	-	-	-
	Dog	0.2	1	-	-	-
	Opossum	1	0.1	0.2	-	-
	Platypus	0.02	3×10^{-5}	8×10^{-5}	0.06	-
	Chicken	2×10^{-11}	4×10^{-10}	2×10^{-11}	3×10^{-9}	5×10^{-11}
LINEs	Mouse	0.2	-	-	-	-
	Dog	1	0.5	-	-	-
	Opossum	0.9	0.02	0.8	-	-
	Platypus	1	0.01	1	1	-
	Chicken	6×10^{-5}	0.08	0.001	4×10^{-5}	3×10^{-8}
RNAs	Mouse	1	-	-	-	-
	Dog	1	0.2	-	-	-
	Opossum	0.6	0.04	1	-	-
	Platypus	0.8	0.09	1	1	-
	Chicken	0.08	0.005	0.2	0.8	1
Total repeats	Mouse	0.06	-	-	-	-
	Dog	0.3	1	-	-	-
	Opossum	1	0.1	0.3	-	-
	Platypus	1	0.06	0.3	1	-
	Chicken	4×10^{-13}	4×10^{-10}	3×10^{-7}	8×10^{-8}	9×10^{-12}

p-values from pairwise t-tests comparing proportion of sequence in each repeat class between each species in the whole genome

Table S2B

Repeat family		Human	Mouse	Dog	Opossum	Platypus
LTRs	Mouse	$2x10^{-16}$	-	-	-	-
	Dog	$2x10^{-16}$	$2x10^{-16}$	-	-	-
	Opossum	$2x10^{-16}$	$2x10^{-16}$	$2x10^{-16}$	-	-
	Platypus	$2x10^{-16}$	$2x10^{-16}$	$2x10^{-16}$	$2x10^{-16}$	-
	Chicken	$2x10^{-16}$	$2x10^{-16}$	$2x10^{-16}$	$2x10^{-16}$	$2x10^{-16}$
DNA elements	Mouse	$2x10^{-16}$	-	-	-	-
	Dog	$2x10^{-16}$	$2x10^{-16}$	-	-	-
	Opossum	$2x10^{-16}$	$2x10^{-16}$	$2x10^{-16}$	-	-
	Platypus	$2x10^{-16}$	0.005	$2x10^{-16}$	$2x10^{-16}$	-
	Chicken	$2x10^{-16}$	$5x10^{-13}$	$2x10^{-16}$	$2x10^{-16}$	$2x10^{-16}$
Simple repeats	Mouse	$2x10^{-16}$	-	-	-	-
	Dog	$2x10^{-16}$	$2x10^{-16}$	-	-	-
	Opossum	$2x10^{-16}$	$2x10^{-16}$	$2x10^{-16}$	-	-
	Platypus	$2x10^{-16}$	$2x10^{-16}$	$2x10^{-16}$	$2x10^{-16}$	-
	Chicken	$2x10^{-16}$	$2x10^{-16}$	$2x10^{-16}$	$2x10^{-16}$	0.2
Low complexity sequence	Mouse	$2x10^{-16}$	-	-	-	-
	Dog	$2x10^{-16}$	$2x10^{-16}$	-	-	-
	Opossum	$2x10^{-16}$	0.002	$2x10^{-16}$	-	-
	Platypus	$2x10^{-16}$	$2x10^{-16}$	$2x10^{-16}$	$2x10^{-16}$	-
	Chicken	0.1	$2x10^{-16}$	$2x10^{-16}$	$2x10^{-16}$	$2x10^{-16}$
SINEs	Mouse	$2x10^{-16}$	-	-	-	-
	Dog	$2x10^{-16}$	$2x10^{-16}$	-	-	-
	Opossum	0.01	$2x10^{-16}$	$2x10^{-16}$	-	-
	Platypus	$2x10^{-16}$	$2x10^{-16}$	$2x10^{-16}$	$2x10^{-16}$	-
	Chicken	$2x10^{-16}$	$2x10^{-16}$	$2x10^{-16}$	$2x10^{-16}$	$2x10^{-16}$
LINEs	Mouse	0.01	-	-	-	-
	Dog	0.1	0.2	-	-	-
	Opossum	$2x10^{-16}$	$2x10^{-16}$	$2x10^{-16}$	-	-
	Platypus	0.0003	$4x10^{-10}$	$7x10^{-10}$	$2x10^{-16}$	-
	Chicken	$2x10^{-16}$	$2x10^{-16}$	$2x10^{-16}$	$2x10^{-16}$	$2x10^{-16}$
RNA	Mouse	$2x10^{-16}$	-	-	-	-
	Dog	$2x10^{-16}$	$2x10^{-16}$	-	-	-
	Opossum	$2x10^{-16}$	$2x10^{-16}$	$2x10^{-16}$	-	-
	Platypus	$2x10^{-16}$	$2x10^{-16}$	$2x10^{-16}$	0.05	-
	Chicken	$2x10^{-16}$	$2x10^{-16}$	$2x10^{-16}$	$2x10^{-16}$	$2x10^{-16}$
Total repeats	Mouse	$2x10^{-16}$	-	-	-	-
	Dog	$2x10^{-16}$	$2x10^{-16}$	-	-	-
	Opossum	$2x10^{-16}$	$2x10^{-16}$	$2x10^{-16}$	-	-
	Platypus	$2x10^{-6}$	$4x10^{-8}$	$2x10^{-16}$	$2x10^{-16}$	-
	Chicken	$2x10^{-16}$	$2x10^{-16}$	$2x10^{-16}$	$2x10^{-16}$	$2x10^{-16}$
